# Adult and paediatric haematology and clinical chemistry laboratory reference limits for Liberia

**DOI:** 10.4102/ajlm.v9i1.1080

**Published:** 2020-11-25

**Authors:** Mark W. Kieh, Sarah M. Browne, Greg A. Grandits, Julie Blie, Jestina W. Doe-Anderson, Marie L. Hoover, Bionca Davis, Cavan S. Reilly, James D. Neaton, H. Clifford Lane, Stephen B. Kennedy

**Affiliations:** 1 Partnership for Research on Ebola Virus in Liberia (PREVAIL), New Kru Town, Monrovia, Liberia; 2 Division of Biostatistics, University of Minnesota, Minneapolis, Minnesota, United States; 3 Leidos Biochemical Research, Fredrick, Maryland, United States; 4 Advanced BioMedical Laboratories, Cinnaminson, New Jersey, United States; 5 Division of Clinical Research, National Institute of Allergy and Infectious Diseases, National Institute of Health, Bethesda, Maryland, United States; 6 Liberian College of Physicians and Surgeons, Monrovia, Liberia

**Keywords:** reference ranges, Liberia, chemistries, haematology, paediatric

## Abstract

**Background:**

As more research is conducted in Liberia, there is a need for laboratory reference limits for common chemistry and haematology values based on a healthy population. Reference limits from the United States may not be applicable.

**Objective:**

The aim of this study was to present laboratory reference ranges from a Liberian population and compare them to United States ranges.

**Methods:**

Serum chemistry and haematology values from 2529 adults and 694 children and adolescents obtained from two studies conducted in Liberia between 2015 to 2017 were used to determine reference limits. After removing outliers, the reference limits defined by the 2.5th and 97.5th percentiles were determined by sex in three age groups (6–11, 12–17, and 18+ years).

**Results:**

The median (interquartile range) of adults was 29 (23, 37) years; 44% were female. The median (interquartile range) for children and adolescents was 12 (9, 15) years; 53% were female. Several reference ranges determined using Liberian participants differed from those in the US. For chemistries, a high percentage of both adults and children/adolescents had high serum chloride levels based on United States ranges. For haematology, a high percentage of Liberian participants had haemoglobin and related assays below the lower limit of United States ranges.

**Conclusion:**

Chemistry and haematology reference intervals determined for a Liberian population of healthy individuals should be considered for establishing eligibility criteria and monitoring of laboratory adverse events for clinical trials as well as for use in clinical settings in Liberia and perhaps for other countries in Western Africa.

## Introduction

Reference limits for clinical laboratory tests for healthy adults and children in Liberia are not available based on a Liberian population. In a clinical setting, reference limits determined from a healthy population can provide useful information for making decisions based on laboratory reports. In addition, in a research setting, reference limits are often used for inclusion and exclusion criteria, for assessing possible adverse effects of treatment, and for the diagnosis of outcomes. Differences in reference limits for haematology and chemistry measurements have been reported between United States (US) reference intervals and reference intervals determined for healthy individuals in other African countries.^1,2,3,4,5,6,7,8,9,10,11,12,13,14^ These differences have been attributed to differences in socioeconomic status, diet, physical exercise, environmental pathogens and altitude.

The Partnership for Research on Ebola Virus in Liberia (PREVAIL) initiated several studies beginning in 2015. Our experience carrying out this research motivated the determination of reference limits based on healthy people living in Liberia who enrolled in two of the studies. For example, in one of these studies, a vaccine trial for the prevention of Ebola virus disease (EVD), a large percentage of apparently healthy participants had initial laboratory test result values outside the limits considered ‘normal’.

The purpose of this paper is three-fold: (1) to describe chemistry and haematology test results for a large number of apparently healthy adults and children in Liberia; (2) to use that data to define reference limits for use in future research projects in Liberia; and (3) to compare the reference limits determined based on Liberian participants with those based on US participants.

## Methods

### Ethical considerations

We used data from two studies. PREVAIL I and PREVAIL III, to determine the reference limits. All participants aged 18 years and older provided written informed consent. A parent or guardian signed a written informed consent for all participants under age 18 years, and children aged 9 years and older also signed a written assent. Both study protocols were approved by the National Research Ethics Board of Liberia and the Institutional Review Board of the United States National Institutes of Health; Protocol identification number 15-I-N071 (PREVAIL I) and 15-I-0122 (PREVAIL III).

### Study design and sample selection

The PREVAIL I study was a Phase 2 placebo-controlled randomised trial that evaluated the efficacy and safety of two vaccines to prevent EVD. The PREVAIL III study was a cohort study of EVD survivors and their close contacts. Close contacts were identified by survivors and either lived with the survivor at the time of diagnosis or after discharge from the Ebola treatment unit, or were sexual partners after discharge. The study design, methods and results of PREVAIL I and PREVAIL III have been described elsewhere.^15,16,17^

### Study participants used to determine reference limits

In PREVAIL I, volunteers aged 18 years or older were enrolled over a three-month period beginning in February 2015 at Redemption Hospital in Monrovia, Liberia. The trial excluded participants with a history of EVD, those with a temperature of more than 38 ºC, and women who were pregnant or breast-feeding. The following additional exclusions were made for these analyses: (1) participants with a history of high blood pressure, diabetes or cancer; (2) participants with HIV or syphilis infection based on blood testing; and (3) participants with antibody levels against the Ebola virus surface glycoprotein greater than or equal to 548 enzyme-linked immunosorbent assay units (EU)/mL which was considered indicative of past Ebola infection. These additional exclusions were made to remove potentially unhealthy participants that could have abnormal laboratory values as a result of medical conditions.

In PREVAIL III, close contacts of EVD survivors of any age, identified by EVD survivors, were enrolled between 2015 and 2017. Close contacts were enrolled at three sites: John F. Kennedy (JFK) Medical Centre and Duport Road Clinic (both in Monrovia), and C.H. Rennie Hospital (a more rural site about 70 km north of Monrovia).

For participants in PREVAIL III, the following additional exclusions were made for these analyses: (1) participants with a history of high blood pressure, diabetes, cancer, stroke or ischemic heart disease; (2) participants with HIV or syphilis based on blood testing; and (3) participants with antibody levels against the Ebola virus surface glycoprotein greater than or equal to 548 EU/mL.

**FIGURE 1:**
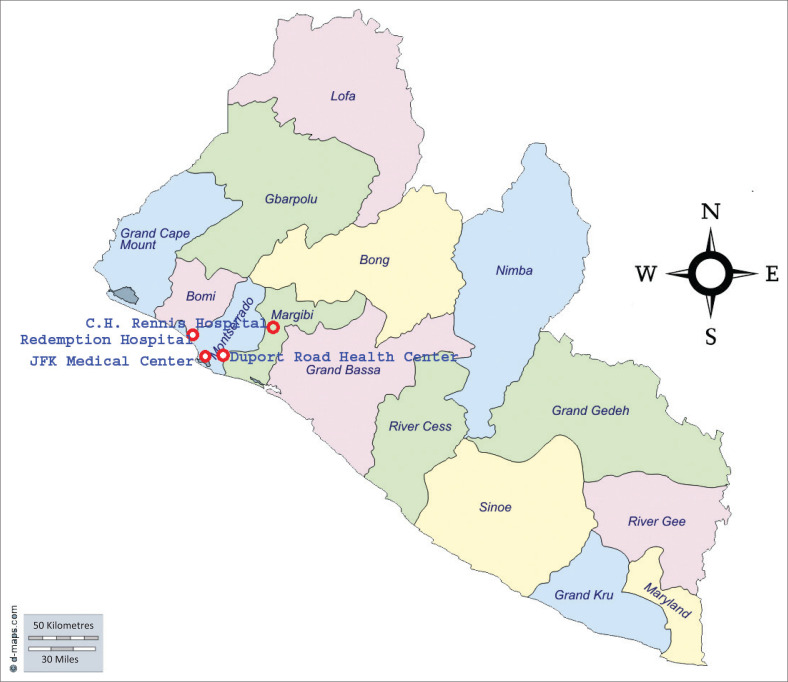
Map of Liberia and location of study sites.

A map showing the locations of the sites where participants were enrolled in PREVAIL I and III is provided in Figure 1. Monrovia is a coastal city located on the Atlantic Coast, with elevation just above sea level.

### Laboratory measurements

Laboratories were established at Redemption Hospital, JFK Medical Centre, and C.H. Rennie Hospital. For participants enrolled in PREVAIL I, all laboratory testing was done at Redemption Hospital. For participants enrolled in PREVAIL III, the testing was done at JFK Hospital (JFK participants) or C.H. Rennie Hospital (C.H. Rennie and Duport Road participants), with occasional backup testing at Redemption Hospital. The same laboratory equipment and methods for blood drawing, specimen labelling and testing were used by all three laboratories, with written documentation of all procedures provided in Standard Operating Procedures. All samples were typically analysed within 4 h. Calibrators and controls (vender and third-party) were run daily at each site to maintain quality assurance. The vendors provided precision data for each analyte. Calibrators and control samples were run before the study samples, and results needed to be within the accepted range in order for the study samples to be analysed.

In both studies, the participants were seen in the morning for their baseline clinic visits, during which non-fasting venous blood specimens were obtained. Blood was collected in serum separator tubes for chemistry analyses and Ethylenediaminetetraacetic acid tubes for haematology. Specimens were then transferred to the respective laboratory. All samples were accessioned, centrifuged (if required) and then analysed on benchtop instrumentation: haematology using Cell Dyn Ruby (Abbott Diagnostics, Abbott Park, Illinois, United States), and chemistries using Trademark name for Alfa Wassermann’s first chemistry analyzer (ACE) Alera or ACE Axcel (Alfa Wassermann, West Caldwell, New Jersey, United States), with Alera or Axcel used interchangeably. In addition to assessing HIV and syphilis serostatus, a complete blood count was obtained with differential and platelet count, aspirate aminotransferase, alanine aminotransferase, creatinine, potassium, chloride and sodium. Alcohol intake was not ascertained in either study.

Each day reports were generated giving chemistry and haematology results for each participant and indicating values that were outside ‘normal’ limits based on US values.

### Data analysis

Laboratory data for PREVAIL I and III were combined for these analyses. As a first step, outliers for each test were removed after Box-Cox transformation (restricting transformation to the identity, square root or log) using a method proposed by Tukey:^18^ values 1.5 × interquartile range above the 75th percentile or 1.5 × interquartile range below the 25th percentile were removed. This was done because the information collected to exclude participants with chronic conditions was minimal and the goal was to identify a ‘healthy’ population. Following this step, for each laboratory test, the reference limits defined by the 2.5th and 97.5th percentiles were determined based on a commonly used non-parametric approach.^19,20^ Percentile values were back-transformed to the original scale. Because it is important that reference ranges consider age and sex, this process was performed separately for each sex and age group (6–11, 12–17, and 18 years and over). The median and reference ranges are cited for male and female individuals in each of the three age groups. The percentage of participants both below and above published US reference ranges^21^ are cited for each laboratory value. The results found in this study and for other countries in West Africa are given for reader reference. No formal statistical tests were made for these comparisons. Non-parametric tests were conducted for differences in medians between male and female participants and among the three age groups by sex. All analyses were performed using SAS version 9.4 (SAS Institute, Cary, North Carolina, United States). *P*-values < 0.001 were regarded as statistically significant.

## Results

**FIGURE 2:**
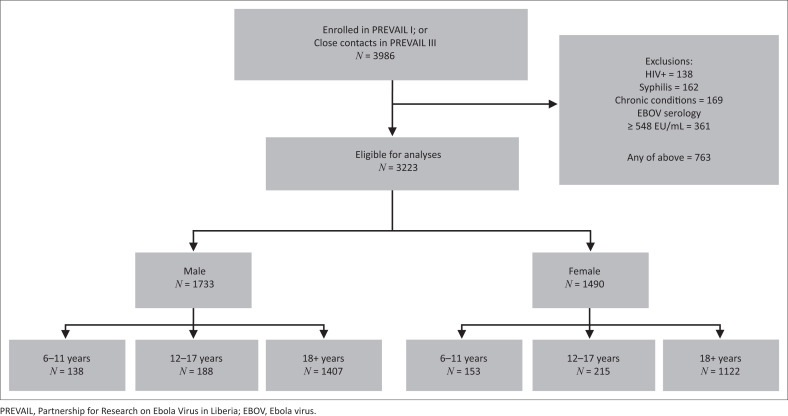
Flow diagram for inclusion of participants for determination of reference levels: Monrovia, Liberia 2015–2017.

Of the 3986 participants enrolled in PREVAIL I and III, 3223 met the eligibility criteria for these analyses (Figure 2). The adults enrolled in PREVAIL I that are included in these analyses had a median age of 29 years, 33% were female, and the median body mass index was 21.6 kg/m^2^ (Table 1). The adults in PREVAIL III that are included in these analyses had similar age distributions as those of the PREVAIL I adults, but included a higher percentage of women and had a slightly higher median body mass index. The median age for the children/adolescents was 12 years, 53% were female, and the median body mass index was 17.4 kg/m^2^.

**TABLE 1:** Baseline characteristics of study participants in laboratory analysis: Monrovia, Liberia 2015–2017.

Age of participants	PREVAIL I			PREVAIL III		
	*N*	*Median or %*	IQR	*N*	*Median or %*	IQR
**Adults 18+ y**						
Age (y)	-	29.0	24.0–36.0	-	29.0	23.0–38.0
Female	418	33.4	-	704	55.1	-
Body mass index kg/m^2^	-	21.6	20.0–24.0	-	23.0	20.9–26.3
No. of participants	1251	-	-	1278	-	-
**Children/adolescents 6–17 y**						
Age (y)	N/A	-	-	-	12.0	9.0–15.0
Female	N/A	-	-	368	53.0	-
Body mass index kg/m^2^	N/A	-	-	-	17.4	15.7–20.0
No. of participants	N/A	-	-	694	-	-

Total number of participants: PREVAIL I =1251, PREVAIL III =1972.

PREVAIL I participants were all adults. Values in cells are either median interquartile range, *N* or %.

PREVAIL, Partnership for Research on Ebola Virus in Liberia; N/A, No children/adolescents were enrolled in PREVAIL I.

IQR, interquartile range; y, years.

### Laboratory median levels and reference limits by age and gender

Many laboratory median levels and corresponding reference ranges differed significantly by sex and age (Table 2). For example, the median and reference ranges for white blood cell (WBC) count in male participants aged 6–11 years was 7.18 (4.61–11.92) x 10^3^/*μ*L compared to 5.77 (3.63–9.72) x 10^3^/*μ*L in adult female participants.

**TABLE 2:** Median and reference ranges for chemistries and haematology by gender and age group: Monrovia, Liberia 2015–2017.

Laboratory analyte	Males						Females					
	Age 6–11		Age 12–17		Age 18+		Age 6–11		Age 12–17		Age 18+	
	Med	Ref range	Med	Ref range	Med	Ref range	Med	Ref range	Med	Ref range	Med	Ref range
**Chemistry**												
ALT IU/L	8.0	3.0–18.0	7.0	2.0–18.0	9.0	2.0–43.0	7.0	2.0–19.0	6.0	2.0–19.0	7.0	1.0–31.0
AST IU/L	21.0	13.0–33.0	18.0	9.0–31.0	15.0	7.0–34.0	20.0	12.0–31.0	14.0	8.0–23.0	12.0	6.0–26.0
Creatinine mg/dL	0.57	0.40–0.78	0.75	0.47–1.09	1.07	0.78–1.49	0.56	0.33–0.79	0.67	0.45–0.92	0.80	0.54–1.10
Chloride mmol/L	105.6	101.3–109.2	104.4	101.3–108.7	104.3	100.2–109.0	105.9	102.4–110.3	105.3	101.7–109.2	106.1	102.2–110.1
Potassium mmol/L	4.22	3.50–5.19	4.30	3.40–5.32	4.25	3.54–5.19	4.17	3.40–5.25	4.20	3.48–5.00	4.10	3.30–5.00
Sodium mmol/L	139.2	136.5–143.0	140.3	137.4–143.3	141.0	137.4–145.2	139.8	137.1–143.4	140.2	136.3–143.3	141.5	137.0–145.9
**Haematology**												
WBC 10^3^/μL	7.18	4.61–11.92	6.13	3.79–9.60	5.17	3.27–8.47	7.14	4.19–12.40	6.56	4.22–10.47	5.77	3.63–9.72
Neutrophils 10^3^/μL	2.63	1.32–5.90	2.19	1.25–4.40	2.21	1.07–4.86	2.59	1.17–5.75	2.55	1.29–6.14	2.57	1.22–5.60
Lymphocytes 10^3^/μL	3.20	1.82–6.13	2.69	1.36–4.53	2.01	1.16–3.46	3.14	1.64–5.51	2.72	1.55–4.30	2.25	1.32–3.99
Monocytes 10^3^/μL	0.62	0.32–1.22	0.52	0.29–1.01	0.43	0.22–0.81	0.61	0.32–1.22	0.52	0.30–0.99	0.41	0.23–0.78
Eosinophils 10^3^/μL	0.41	0.08–2.99	0.32	0.06–1.87	0.22	0.03–1.09	0.28	0.05–2.67	0.25	0.04–1.07	0.18	0.02–0.94
Basophils 10^3^/μL	0.09	0.03–0.20	0.07	0.03–0.15	0.06	0.03–0.14	0.08	0.03–0.19	0.06	0.02–0.13	0.05	0.02–0.12
RBC 10^6^/μL	4.86	3.95–5.92	5.16	4.00–6.47	5.34	4.28–6.53	4.85	3.72–5.90	4.86	4.03–5.70	4.76	3.81–5.81
HGB g/dL	11.80	9.70–13.60	12.54	9.91–15.97	14.44	11.47–16.94	11.90	9.83–14.04	12.31	10.30–14.40	12.64	9.93–15.04
MCV fL	73.65	62.21–84.38	76.50	61.68–85.50	82.31	68.48–92.12	74.34	64.20–83.95	78.60	64.90–87.76	80.90	66.96–91.20
MCH pg	24.56	19.76–29.10	24.97	19.60–28.60	27.40	21.09–31.70	24.81	19.60–28.84	25.91	20.00–29.90	26.80	20.35–31.30
MCHC g/dL	33.30	30.73–35.40	32.68	29.54–35.62	33.20	30.40–35.69	33.02	30.23–35.56	32.70	29.32–35.34	33.08	30.03–35.70
HCT *%*	35.56	30.00–41.30	38.71	31.69–47.73	43.50	35.60–51.10	36.23	29.16–42.18	37.57	30.92–43.36	38.30	31.00–44.80
RDW *%*	13.28	11.40–15.85	13.29	11.57–16.30	12.70	11.10–15.41	13.10	11.60–15.83	12.84	11.30–15.40	12.80	11.20–15.72
Platelets 10^3^/μL	303.0	169.4–557.3	256.5	178.7–373.7	211.6	119.4–325.6	300.5	149.0–483.0	264.7	151.0–402.0	247.0	136.1–376.6
MPV fL	8.31	5.65–12.60	8.22	6.04–12.61	7.84	5.61–11.94	7.95	5.35–12.40	8.12	5.91–11.82	8.02	5.63–11.94
**Number of participants**	**138**	**-**	**188**	**-**	**1407**	**-**	**153**	**-**	**215**	**-**	**1122**	**-**

The number of participants displayed is before the removal of outliers which varies by assay. The number of participants differ slightly among laboratory tests because of missing values.

ALT, alanine aminotransferase; AST, aspartate aminotransferase; HCT, haematocrit; HGB, haemoglobin; MCH, mean corpuscular haemoglobin; MCHC, mean corpuscular haemoglobin concentration; MCV, mean corpuscular volume; Med, median; MPV, mean platelet volume; RBC, red blood cell count; RDW, red cell distribution width; WBC, white blood cell count; Ref, reference.

For chemistry, adult men had higher liver enzymes, creatinine, and potassium levels, with lower serum chloride and sodium levels than adult women (*p* < 0.001). In general, the differences between sexes were less pronounced in children/adolescents. For example, the median serum creatinine levels were nearly identical for male and female individuals aged 6–11 years and only slightly higher for male than female individuals aged 12–17 years. For haematology values, adult men had lower WBC counts but higher red blood cell counts and haemoglobin levels than adult women (*p* < 0.001). Platelet counts were higher in adult women than men. The differences in haematology values between sexes were much smaller in the youngest age group (ages 6–11 years) and were not statistically significant.

For serum chemistry, the median aminotransferase was inversely related to age for both male and female participants, whereas serum creatinine was strongly positively related to age for both sexes (*p* < 0.001). The median serum sodium increased with age for both sexes. For haematology, the WBC counts were highest for ages 6–11 years for both sexes and the red blood cell counts increased with age for male but not female participants. The median haemoglobin levels increased with age in both sexes but the relationship was stronger in male participants. Platelets decreased with age for both male and female participants.

### Comparison with United States reference ranges

For chemistries, a high percentage of both adults and children/adolescents would be classified as having high serum chloride levels based on the US reference standard (Table 3). Similarly, several haematology factors would be classified as out of range for both adults and children/adolescents using the US standard. A high percentage of both children/adolescent and adult participants had haemoglobin, mean corpuscular volume, mean corpuscular haemoglobin, mean corpuscular haemoglobin concentration, and haematocrit levels below the lower limit. In addition, 20% – 25% of Liberian adults had WBC counts and neutrophils below the US reference standard.

**TABLE 3:** Percentage of Study Participants Outside of US1 Reference Intervals: Monrovia, Liberia 2015-2017

Laboratory analyte	Reference Interval (MGH)†	Age 6-17				Age 18+			
		Low Levels		High Levels		Low Levels		High Levels	
		*N*	%	*N*	%	*N*	%	*N*	%
**Chemistries**									
ALT (IU/L)	0–35	N/A	-	0	0.0	N/A	-	69	2.9
AST (IU/L)	0–35	N/A	-	3	0.5	N/A	-	32	1.3
Creatinine (mg/dl)	0–1.5	N/A	-	0	0.0	N/A	-	20	0.8
Chloride (mmol/L)	98–106	0	0.0	247	36.8	0	0.0	835	34.2
Potassium (mmol/L)	3.5–5.0	16	2.3	30	4.4	90	3.6	100	4.0
Sodium (mmol/L)	136–145	4	0.6	0	0.0	12	0.5	97	4.0
**Hematology**									
WBC (103/uL)	4.5–11.0	43	6.3	21	3.1	566	22.7	2	0.1
Neutrophils (103/uL)	1.8–7.7	117	17.1	0	0.0	616	24.6	0	0.0
Lymphocytes (103/uL)	1.0–4.8	0	0.0	36	5.3	4	0.2	2	0.1
Monocytes (103/uL)	0–0.8	N/A	-	104	15.2	0	0.0	58	2.3
Eosinophils (103/uL)	0.45	N/A	-	238	34.6	N/A	-	489	19.6
Basophils (103/uL)	0–0.2	N/A	-	6	0.9	N/A	-	0	0.0
RBC (106/uL)	4.5–5.9; 4.0–5.2	67	9.8	98	14.4	155	6.2	463	18.5
HGB (g/dL)	13.5–17.5; 12.0–16.0	426	62.7	0	0.0	648	26.2	10	0.4
MCV (fl)	80–100	532	77.6	0	0.0	969	39.3	0	0.0
MCH (pg)	26–34	432	63.3	0	0.0	845	34.0	4	0.2
MCHC (g/dL)	31–37	68	10.1	0	0.0	198	8.0	1	0.0
HCT (%)	41–53; 36–46	389	57.7	0	0.0	635	25.7	12	0.5
RDW (%)	11.5–14.5	22	3.2	107	15.8	180	7.3	235	9.6
Platelets (103/uL)	150–350	9	1.3	111	16.6	191	7.7	74	3.0
MPV (fl)	6-9–10.6	137	20.3	75	11.1	597	24.1	226	9.1
**Number of Participants**	**-**	**694**	**-**	**-**	**-**	**2529**	**-**	**-**	**-**

The number of participants displayed is before removal of outliers which varies by assay. Actual number of participants ranged from 635-687 for ages 6-17 and 2387-2498 for ages 18+.

ALT, alanine aminotransferase; AST, aspartate aminotransferase; HCT, hematocrit; HGB, hemoglobin; MCH, mean corpuscular hemoglobin; MCHC, mean corpuscular hemoglobin concentration; MCV, mean corpuscular volume; MPV, mean platelet volume; RBC, red blood cell count; RDW, red cell distribution width; WBC, white blood cell count; N/A, Not applicable, no lower limit.

† , Based on Massachusetts General Hospital (MGH). When two ranges are given the first is for males and the second for females. Numbers for low (below lower limit) and high (above upper limit) are N (%).

### Reference ranges for other West African countries

Medians and reference limits for our Liberian study showed some differences compared to reference limits reported for Ghana, Mali, and Gambia (Table 4);^4,10,13,14^ chemistry values were only available from Ghana. Liver enzymes were lower in Liberia than in Ghana. Potassium, sodium, and chloride levels were also lower in the Liberian population than in the Ghanaian population. The median and reference limits for WBC counts were similar for men in Liberia and Mali. The median WBC counts for women in Liberia were higher than in the other West African countries. The red blood cell counts for men in Liberia were similar to both Ghana and Mali, while the red blood cell counts for women in Liberia were higher than those in Ghana. The haemoglobin levels were similar in the Liberian population as compared to the other West African countries. Platelet counts differed among countries but were always higher in women than men in each country. Comparison of medians among countries were not always consistent with the reference ranges, suggesting differences in variability between populations.

**TABLE 4:** Comparisons of adult Liberian reference ranges with other adult African countries reference ranges, Monrovia, Liberia 2015–2017.

Laboratory analyte	Liberian adults our study				Ghana Dosoo 2012, Addai-Mensah 2019^13,14^				Mali Kone 2017^4^				Gambia Adetifa 2008^10^	
	Male		Female		Male		Female		Male		Female		Male	Female
	Med	REF range	Med	REF range	Med	REF range	Med	REF range	Med	REF range	Med	REF range		
**Chemistries**														
ALT IU/L	9	2–43	7	1–31	23	8–54	17	6–51	N/A	N/A	N/A	N/A	N/A	N/A
AST IU/L	15	7–34	12	6–26	30	17–60	23	13–48	N/A	N/A	N/A	N/A	N/A	N/A
Creatinine mg/dL	1.07	0.78–1.49	0.80	0.54–1.10	0.96	0.63–1.35	0.84	0.53–1.24	N/A	N/A	N/A	N/A	N/A	N/A
Chloride mmol/L	104	100–109	106	102–110	107	101–115	108	102–113	N/A	N/A	N/A	N/A	N/A	N/A
Potassium mmol/L	4.3	3.5–5.2	4.1	3.3–5.0	4.5	3.6–5.2	4.3	3.4–5.1	N/A	N/A	N/A	N/A	N/A	N/A
Sodium mmol/L	141	137–145	142	137–146	144	135–151	145	135–150	N/A	N/A	N/A	N/A	N/A	N/A
**Haematology**														
WBC 10^3^/μL	5.2	3.3–8.5	5.8	3.6–9.7	5.5	3.3–11.2	5.6	3.3–10.6	5.2	3.1–11.1	4.7	3.8–12.5	3.3–8.2	3.5–8.4
Neutrophils 10^3^/μL	2.21	1.07–4.86	2.57	1.22–5.60	2.08	0.65–5.50	2.23	0.57–6.08	2.2	1.0–4.4	2.2	1.2–7.4	N/A	N/A
Lymphocytes 10^3^/μL	2.01	1.16–3.46	2.25	1.32–3.99	2.35	0.77–4.78	2.25	0.64–4.28	2.1	1.2–3.8	2.2	1.4–4.6	N/A	N/A
Monocytes 10^3^/μL	0.43	0.22–0.81	0.41	0.23–0.78	0.51	0.21–1.02	0.48	0.19–1.02	0.2	0.1–0.7	0.2	0.1–0.5	N/A	N/A
Eosinophils 10^3^/μL	0.22	0.03–1.09	0.18	0.02–0.94	0.16	0.01–0.90	0.12	0.02–0.90	0.17	0–1.03	0.07	0–0.83	N/A	N/A
Basophils 10^3^/μL	0.06	0.03–0.14	0.05	0.02–0.12	0.03	0.01–0.09	0.03	0.01–0.11	0.04	0–0.11	0.00	0–0.11	N/A	N/A
RBC 10^6^/μL	5.3	4.3–6.5	4.8	3.8–5.8	5.2	3.6–7.0	4.4	3.1–5.9	5.1	4.2–6.21	4.7	3.9–5.8	N/A	N/A
HGB g/dL	14.4	11.5–16.9	12.6	9.9–15.0	15.2	10.7–18.8	12.5	8.2–16.2	14.5	12.4–17.6	12.8	12.0–14.9	11.1–16.6	9.8–15.0
MCV fL	82	68–92	81	67–91	87	70–103	87	64–104	87	72–98	86	39–118	74–95	72–94
MCH pg	27	21–32	27	20–31	29	23–34	29	20–34	29	23–34	28	23–35	N/A	N/A
MCHC g/dL	33.2	30.4–35.7	33.1	30.0–35.7	33.7	29.7–37.2	33.1	26.8–37.1	33.0	30.9–34.9	32.5	30.9–34.5	N/A	N/A
HCT %	43.5	35.6–51.1	38.3	31.0–44.8	45.2	31.8–61.8	37.7	26.8–50.4	43.5	33.2–54.6	39.5	26.8–52.5	N/A	N/A
RDW %	12.7	11.1–15.4	12.8	11.2–15.7	14.0	11.7–18.7	14.3	11.8–26.4	13.4	11.9–17.1	13.7	11.6–24.3	N/A	N/A
Platelets 10^3^/μL	211	119–325	247	136–377	186	86–348	214	111–416	259	133–460	291	151–532	124–367	140–397
MPV fL	7.8	5.6–11.9	8.0	5.6–11.9	-	-	-	-	7.8	5.7–9.4	8.0	6.0–10.5	N/A	N/A
**Number participants**	**1407**	**-**	**1122**	**-**	**216**	**-**	**266**	**-**	**173**	**-**	**40**	**-**	**599**	**680**

Values for Gambia are 90% reference intervals; all other intervals are 95%.

ALT, alanine aminotransferase; AST, aspartate aminotransferase; HCT, hematocrit; HGB, hemoglobin; MCH, mean corpuscular hemoglobin; MCHC, mean corpuscular hemoglobin concentration; MCV, mean corpuscular volume; Med, median; MPV, mean platelet volume; RBC, red blood cell count; RDW, red cell distribution width; Ref range, reference range; WBC, white blood cell count; N/A, not available.

## Discussion

To determine reference limits for common clinical laboratory test results for people in Liberia by age and sex, we used baseline data collected at the time of enrolment in two research studies conducted during the West African Ebola epidemic. One study enrolled healthy adults in a vaccine trial^16^ and the other enrolled adults and children who were close contacts of individuals who survived Ebola.^17^ ‘Unhealthy’ participants were excluded based on medical history and for positive HIV or syphilis tests. Our goal was to select individuals for determining reference intervals and to use recommended statistical methods for determining these intervals as outlined by the Clinical and Laboratory Standards Institute^22^ that could be used to interpret an individual’s laboratory test results and could be used in the design of future clinical research studies. We found that for several laboratory tests, the reference limits based on the data from this Liberian population differed greatly from the US-based reference limits. For example, the haemoglobin reference ranges were much lower in this study than the US-based reference values. Based on the US reference limits, one could imply that a high proportion of Liberians have ‘abnormal’ low haemoglobin levels. Other reference limits that differed considerably were chloride, for which the Liberian population had higher reference limits, and mean corpuscular volume, mean corpuscular haemoglobin, haematocrit and mean platelet volume, for which the Liberian population had lower reference limits. Differences between values found in this study compared to US levels could be due to several reasons, including genetics, environmental factors, subclinical disease, and laboratory equipment and/or methods. The observed differences are unlikely to be because of laboratory differences, as the laboratories set up in Liberia used standard equipment that is also used in the US for chemistries and haematology.

The reference limits from our study also confirm that separate values for many laboratory parameters should be considered for men and women and for adults and children/adolescents. The adult women in our study had significantly higher WBC counts than men. The children/adolescents also had higher WBC counts, lower creatinine levels, and lower haemoglobin levels than adults.

Many clinical trials use reference limits and tables for grading laboratory toxicities, such as the Division of AIDS Table for Grading the Severity of Adult and Paediatric Adverse Events,^23^ to define eligibility criteria and to grade adverse events during follow-up. The difference in reference limits between those estimated for Liberia and US limits could impact the number of participants found to be eligible for a trial, as well as the percentage developing adverse events based on laboratory test results. For trials conducted in other parts of Africa this has been the case. Eller et al.^9^ studied the impact of using US reference limits in Uganda to screen participants for an HIV vaccine trial. They found that US reference limits led to more exclusions during screening for a Phase 1 vaccine trial than the use of their reference limits derived from people living in Uganda. They also noted that the Division of AIDS toxicity table did not reflect locally established reference limits, and the lower limit for neutrophils they had estimated would qualify as a grade 2 adverse event. Segolodi et al.^7^ reported that many healthy volunteers for an HIV pre-exposure prophylaxis trial conducted in Botswana had abnormal amylase results according to US-derived reference values. Zeh et al.^24^ reported that over 58% of participants would have been excluded from a trial in Kenya using US reference limits as compared to reference limits determined for the local population. In addition, 40% of otherwise healthy study participants would have been considered to have a grade 1–4 laboratory-based adverse event based on the Division of AIDS toxicity table.

There may be pros and cons to using Liberian versus US reference limits for reporting adverse events. The burden associated with reporting lower-severity grade events,^25^ particularly those based solely on laboratory results and not associated with symptoms, would suggest using reference limits from local populations. On the other hand, in early phase research of novel treatments such as the Ebola studies on which this research is based, it may be more prudent to use more conservative limits such as the US reference limits until safety is established.

A strength of this study is the large number of participants (> 2500 adults and nearly 700 children/adolescents) studied with common laboratory methods in two research studies, allowing for more precise estimates of laboratory percentiles on which the reference limits are based. To our knowledge, this is the first report of laboratory reference limits from a Liberian population. We recommend that additional laboratory-based studies be conducted to establish suitable laboratory reference limits for Liberia.

### Limitations

A limitation to this study is that we based our definition of ‘healthy’ participants largely on a self-reported medical history. While we used statistical methods that attempted to remove outliers to establish a ‘healthy’ population, and that typically removed about 1% – 3% of participants from the group where normal ranges were calculated using 2.5% and 97.5% percentiles, our cohort is likely to include some participants with undiagnosed illnesses.

### Conclusion

In conclusion, we present the chemistry and haematology reference intervals from a Liberian population of healthy individuals for men and women and for children/adolescents and adults. These levels should be considered for screening and monitoring participants in clinical trials in Liberia and perhaps other countries in West Africa.
